# Imaging Techniques in Alzheimer’s Disease: A Review of Applications in Early Diagnosis and Longitudinal Monitoring

**DOI:** 10.3390/ijms22042110

**Published:** 2021-02-20

**Authors:** Wieke M. van Oostveen, Elizabeth C. M. de Lange

**Affiliations:** 1 Faculty of Science, Leiden University, Einsteinweg 55, 2333 CC Leiden, The Netherlands; w.m.van.oostveen@umail.leidenuniv.nl; 2 Division of Systems Biomedicine and Pharmacology, Leiden Academic Centre of Drug Research, Leiden University, Einsteinweg 55, 2333 CC Leiden, The Netherlands

**Keywords:** Alzheimer’s disease, imaging techniques, early diagnosis, longitudinal monitoring, amyloid-β, tau, MRI, PET

## Abstract

Background. Alzheimer’s disease (AD) is a progressive neurodegenerative disorder affecting many individuals worldwide with no effective treatment to date. AD is characterized by the formation of senile plaques and neurofibrillary tangles, followed by neurodegeneration, which leads to cognitive decline and eventually death. Introduction. In AD, pathological changes occur many years before disease onset. Since disease-modifying therapies may be the most beneficial in the early stages of AD, biomarkers for the early diagnosis and longitudinal monitoring of disease progression are essential. Multiple imaging techniques with associated biomarkers are used to identify and monitor AD. Aim. In this review, we discuss the contemporary early diagnosis and longitudinal monitoring of AD with imaging techniques regarding their diagnostic utility, benefits and limitations. Additionally, novel techniques, applications and biomarkers for AD research are assessed. Findings. Reduced hippocampal volume is a biomarker for neurodegeneration, but atrophy is not an AD-specific measure. Hypometabolism in temporoparietal regions is seen as a biomarker for AD. However, glucose uptake reflects astrocyte function rather than neuronal function. Amyloid-β (Aβ) is the earliest hallmark of AD and can be measured with positron emission tomography (PET), but Aβ accumulation stagnates as disease progresses. Therefore, Aβ may not be a suitable biomarker for monitoring disease progression. The measurement of tau accumulation with PET radiotracers exhibited promising results in both early diagnosis and longitudinal monitoring, but large-scale validation of these radiotracers is required. The implementation of new processing techniques, applications of other imaging techniques and novel biomarkers can contribute to understanding AD and finding a cure. Conclusions. Several biomarkers are proposed for the early diagnosis and longitudinal monitoring of AD with imaging techniques, but all these biomarkers have their limitations regarding specificity, reliability and sensitivity. Future perspectives. Future research should focus on expanding the employment of imaging techniques and identifying novel biomarkers that reflect AD pathology in the earliest stages.

## 1. Introduction

Alzheimer’s disease (AD) is a progressive neurodegenerative disorder resulting in memory loss, cognitive impairment, behavioural changes and eventually death [1]. AD is the most common cause of dementia and is predicted to affect more than 152 million people in 2050 [2]. The disease is neuropathologically characterized by the deposition of abnormal protein resulting in the formation of extracellular senile plaques and intracellular neurofibrillary tangles (NFTs) [3,4]. The senile plaques contain primarily neurotoxic amyloid-β (Aβ) [5], whereas NFTs consist of abnormal hyperphosphorylated tau aggregates [6,7]. Although the contribution of abnormal protein deposition to AD is recognized, the exact pathogenesis of AD is complex [8], and definitive diagnosis can only be assured post-mortem by histology staining of the brain [9]. Currently, AD is the only cause of death in the top ten deaths globally for which no effective therapeutic treatment is available, and there are no registered drugs to slow down disease progression [10]. Therefore, much effort is put into understanding the pathogenesis of AD for the development of therapeutic agents [11].

In AD, neuropathological changes occur up to thirty years before clinical manifestation of the disease [12]. The initial pathological event in AD is Aβ deposition, which contributes to the formation of senile plaques. Likewise, hyperphosphorylation results in NFTs, leading to neuronal loss, brain atrophy, neurotoxicity, and ultimately cognitive decline [7]. In 1991, Braak and Braak characterized the spread of NFTs across the brain and defined six different stages [3]. These Braak stages correspond with the expansion of NFTs from transentorhinal regions (stage I/II) to limbic areas (stage III/IV) and neocortical regions (stage V/VI) as AD progresses. 

**Figure 1:**
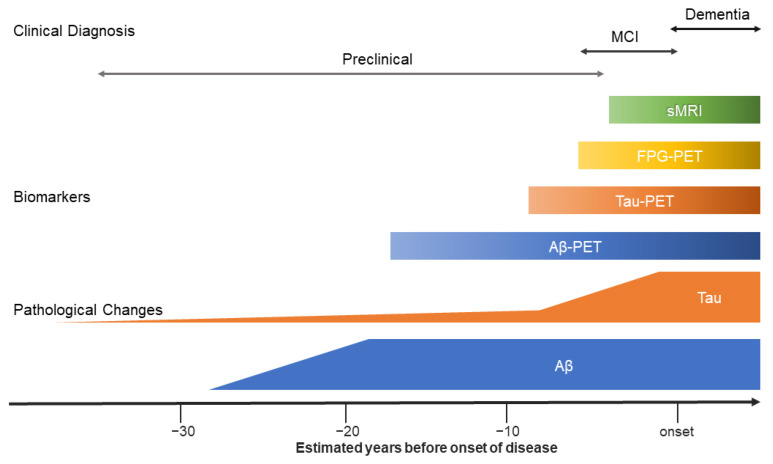
The Alzheimer’s disease continuum with corresponding pathological changes, biomarkers and clinical diagnosis. Figure adapted from Yoshiyama et al. [21].

The above listed events succeed and overlap each other and, therefore, AD is seen as a continuum with pathological changes and clinical symptoms corresponding to the disease stage [13] (Figure 1). Since damage inflicted by these events can surpass a certain neuropathological threshold beyond which any treatment will be unsuccessful, it has been suggested that therapeutic agents should focus on halting neurodegeneration in the silent phase of AD before it becomes too severe [14,15,16]. Therefore, sensitive and specific methods are needed to diagnose AD in the early or preclinical stage [1]. Nowadays, the field of research focuses on identifying so-called biomarkers, which are physiological, chemical or anatomical parameters called biomarkers that effectively reflect certain pathopsychological processes in AD [17]. These biomarkers can be categorized into three different classes based on the type of pathophysiology the biomarker tracks. In this so-called “A/T/N” system, “A” refers to biomarkers measuring Aβ deposition, “T” indicates biomarkers sensitive for tau and “N” the value of biomarkers perceptive for neurodegeneration [18]. This framework is adaptable and can continuously be expanded if new biomarkers become available [19].

An ideal biomarker is inexpensive, easy to monitor and non-invasive and, therefore, will barely harm a patient. Moreover, a good biomarker has high sensitivity and predictive qualities for the specific pathological event [15]. Eventually, biomarkers could offer a diagnostic tool to detect the disease in early stages, thereby providing the opportunity to delay disease progression or even impede the clinical manifestation of the disease [20]. Additionally, monitoring these biomarkers over time could give insight into disease progression and be utilized to track the effectiveness of disease-modifying therapeutics. 

In this review, we focus on biomarkers that can be tracked with structural or functional neuroimaging techniques, such as magnetic resonance imaging (MRI) and positron emission tomography (PET), respectively. The aim of this review is to give an overview of established biomarkers for the early diagnosis and longitudinal monitoring of AD and discuss their feasibility and potential drawbacks. First, we shed light on current biomarkers for the early diagnosis of AD and longitudinal monitoring of disease progression. These biomarkers are reviewed based on their diagnostic utility, benefits and limitations. Subsequently, we introduce new biomarkers and applications of imaging techniques that show promising results for the early diagnosis or longitudinal monitoring of Alzheimer’s disease. Finally, we summarize our findings and provide future perspectives. 

## 2. Contemporary Early Diagnosis of AD with Imaging Techniques

Since the number of patients with AD is increasing due to an aging population, much effort has been put into the detection of the disease as early as possible. Many methods have been tested, ranging from cognitive tests, MRI scans and sampling of cerebral fluid [22]. In this section, we focus on what imaging techniques and associated biomarkers are applied in the early diagnosis of AD.

The first applications of imaging techniques in AD were computed topography (CT) and MRI, but these techniques were used to exclude other causes of dementia rather than to diagnose AD in an early stage [23]. Later, imaging techniques were utilized as positive support to confirm the clinical diagnosis of AD. These techniques focused on the neuronal injury and degeneration aspects of AD [1]. Nowadays, imaging modalities focus on either identifying amyloid deposition or identifying neurodegeneration [24].

### 2.1. Structural MRI

#### 2.1.1. Background

**Figure 2:**
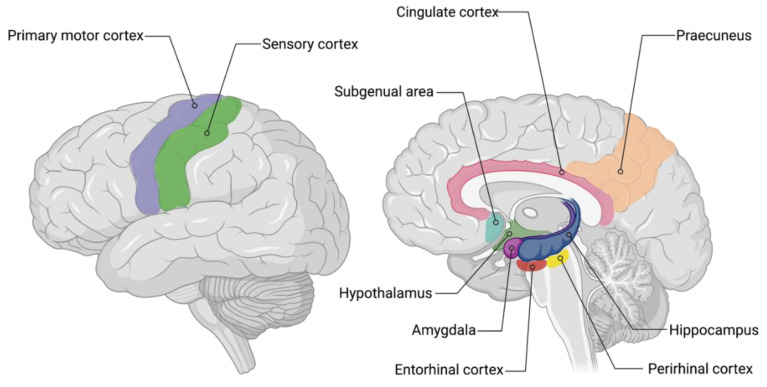
Regions affected by Alzheimer’s disease. Figure created with www.BioRender.com (accessed on 12 February 2021).

The pathology of AD follows a typical spreading pattern through the brain in which certain areas are among the first affected, while other regions will only be impaired in severe stages of AD [3,25]. In this so-called topographic pattern that characterizes AD, the earliest changes are found in the medial temporal lobe structures, the entorhinal and perirhinal cortex and the hippocampus [16] (Figure 2). This typical pattern of disease progression opened possibilities for the early diagnosis of AD by investigating these brain parts with imaging techniques. 

#### 2.1.2. Findings

**Figure 3:**
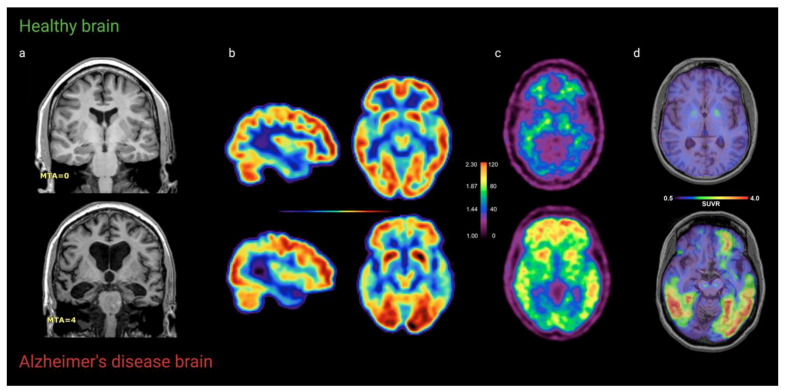
Neuroimages of the healthy versus the Alzheimer’s disease (AD) brain. Neuroimaging with (**a**) structural MRI, (**b**) FDG-PET, (**c**) amyloid-PET with PiB and (**d**) tau PET with ^18^F-AV1451 in both healthy and AD brains. Figure created with www.BioRender.com (accessed on 14 February 2021).

Since neuronal damage in the hippocampus is manifested as decreased hippocampal volume [26], a widely accepted method for assessing AD pathology is volumetric MRI scans of the hippocampus (Figure 3) [25]. These scans are T1-weighted images from which hippocampal atrophy can be measured with either manual or automated segmentation [27]. According to a study of Bobinski et al., MRI provided a powerful tool in assessing the hippocampal volume and predicted volumes that correlated strongly with neuronal numbers, suggesting the anatomic validity of volumetric MRI measurements [28]. Moreover, another study found that volume reductions in the hippocampus are early indications for AD pathology, measurable with MRI [29]. 

In addition to the hippocampus, other limbic brains regions that can be studied with MRI are the entorhinal cortex and amygdala (Figure 2). Although it is believed that the entorhinal cortex is among the regions affected first in AD [30,31,32] and the accuracy of entorhinal cortex volumetry being slightly higher [32], several cross-sectional studies suggested that entorhinal cortex measurements are unlikely to offer additional benefits over hippocampal volumetry in AD patients when compared to healthy controls [33,34,35]. Moreover, high variability in methods to assess the entorhinal cortex volume due to anatomic ambiguity in the cortex’s boundaries eliminates the slight superiority of the entorhinal cortex over the hippocampus [29,34,36]. 

In addition to the use of structural MRI in assessing volume reductions, another application of this imaging technique is to detect cortical thickness reduction in certain brain areas, such as the temporal, orbitofrontal and parietal regions [37]. Detailed study has demonstrated the effect of AD on cortical thickness and led to the suggestion of a so-called AD “disease signature” in which certain brain regions known to be affected by AD show cortical thinning [38]. Assessment of the cortical thickness is believed to be a useful biomarker in the early diagnosis of AD, since subtle changes in areas known to be affected by AD can be detected [39]. Furthermore, a study into region- and phase-specific changes has linked disease severity to cortical thickness [40], thereby coupling the clinical dementia ranking stages to a level of cortical thinning. Additionally, cortical thickness correlates strongly with cognitive impairment in the clinical stages of AD [41,42]. 

Over time, volumetric MRI of the hippocampus has been seen to be the best-established biomarker for AD [1,33,36,43], especially as a diagnostic marker in the mild cognitive impairment stage (MCI) [44]. Additionally, one major benefit of MRI is the availability of appliances in hospitals and research centres [23]. Moreover, MRI is safe and is seen as non-invasive, since it involves no ionizing radiation.

#### 2.1.3. Limitations

However, structural MRI as an imaging technique for AD has its limitations. First, decreased hippocampal volume is not an AD-specific measure [14]. An extensive study by Geuze et al. reviewed more than 420 records reporting the assessment of hippocampal volume with MRI [45]. In addition to AD, other neurodegenerative diseases are characterized with diminished hippocampal volume as well such as Parkinson’s disease [46], epilepsy [47] and Huntington’s disease [48]. Additionally, volume reduction has also been observed after cardiac arrest [49], chronic alcohol abuse [50] and survivors of low birth weight [51]. Moreover, recent study has demonstrated that hippocampal texture predicts conversion from MCI to AD with higher accuracy than the hippocampal volume, although these results have to be validated with histological data [52]. Lastly, manual segmentation of T1-weighted images is time-consuming [53], requires specialistic training and can result in high levels of variability in the measurements [54], due to different protocols for assessing the measurements [33]. Fortunately, in the last decade, much effort has been put into establishing methods for automated segmentation, resulting in more accurate data from MRI images in less time [53,55,56,57]. One major drawback of structural MRI in general is the impossibility to directly observe the effect of amyloid plaques or NFTs in the brain. Atrophy is downstream of the pathological event and not disease specific [23]. Moreover, several studies demonstrated that in atypical forms of AD, the hippocampus is spared [58,59]. Therefore, structural MRI in atypical manifestations of AD might be not able to identify the disease in an early stage. 

### 2.2. FDG-PET

#### 2.2.1. Background

Multiple diseases affecting the central nervous system (CNS) are associated with impaired glucose uptake by neurons [60]. With fluorodeoxyglucose positron emission tomography (FDG-PET), it is possible to measure the resting state cerebral metabolic rates of glucose as a proxy of neuronal activity, without the requirement of cognitive activity [61,62]. FDG-PET measures the uptake of a radiolabeled glucose analogue which correlates with cerebral metabolism and synaptic activity (Figure 3) [23,43]. Since reduced cerebral metabolism is associated with age, healthy age-matched individuals show corresponding cerebral metabolism patterns [62]. The comparison of FDG-PET scans of AD patients with healthy individuals of the same age revealed patterns of metabolic abnormalities in AD, leading to a so-called FDG-PET endophenotype [23]. This endophenotype is seen as a characteristic of AD in which certain brain regions or areas are affected in a spatial pattern [24]. In AD, hypometabolism occurs first in the temporoparietal areas of the brain, including the precuneus and posterior cingulate cortex [1,61] (Figure 2). Moreover, as the disease progresses, the metabolic deficits are gradually aggravated [23].

#### 2.2.2. Findings

Among the first studies that successfully applied FDG-PET in studying Alzheimer’s disease was a research project by Benson et al. in 1983 in which both AD patients and patients with multi-infarct dementia were studied [63]. The results from this study revealed that in AD patients, almost all brain areas demonstrate reduced glucose metabolism, but the primary motor and sensory cortex are spared. This work inspired other researchers and led to an increase in studies investigating the effect of AD on glucose metabolism in the brain [64,65,66]. However, all these studies used patients with diagnosed AD in mild to severe stages of the disease and did not use FDG-PET to diagnose the patients. 

In the 1990s, automated methods to standardize the evaluation of PET scans increased, leading to more consistency in the evaluation of FDG-PET images obtained in different research centers or with different equipment [67,68]. A large study by Silverman et al. used FDG-PET as a diagnostic tool for differentiating healthy individuals from patients with AD symptoms. In the study, the sensitivity and specificity of FDG-PET were addressed, in which sensitivity reflects the ability to identify AD subjects among all individuals, whereas specificity addresses the ability to correctly identify subjects as non-AD. FDG-PET was able to detect AD subjects with a sensitivity of 94% and a 73% specificity. Additionally, in patients diagnosed with questionable or mild dementia, the sensitivity was 95% with a specificity of 71% [69]. These results indicated that FDG-PET is a sensitive indicator of AD and can also be used to assess early-stage dementia. The findings were underlined with other research studies with sensitivity ranging from 84% to 93% and specificity between 63% and 74% [70,71]. Furthermore, reviews based on meta-analyses of articles regarding the identification of AD patients among healthy individuals resulted in pooled sensitivities up to 96% with specificities up to 90% [72,73,74]. Finally, Panegyres et al. demonstrated that FDG-PET is able to differentiate between different types of dementia up to 95% [60]. 

Over the years, FDG-PET emerged to be a relevant and highly specific biomarker for the early diagnosis of AD and other types of major neurodegenerative diseases [43,75]. It is seen as a robust and reliable biomarker in the in vivo diagnosis of early stages of AD [23,36,43]. Moreover, compared to structural MRI of the hippocampus and entorhinal cortex, FDG-PET is diagnostically superior to volumetry measures [76]. Additionally, according to the hypothetical model of dynamic biomarkers proposed by Jack and colleagues, abnormal FDG-PET precedes changes detectable with MRI [77,78] (Figure 1), suggesting an FDG-PET of higher value than structural MRI in the early diagnosis of AD. 

#### 2.2.3. Limitations

However, FDG-PET has its limitations. PET scanners are not widely available and considered as relatively expensive [23,36]. FDG-PET requires the intravenous injection of a radiolabeled agent and is, therefore, more invasive than MRI. Moreover, hypometabolism is a result of neurodegeneration and, therefore, it might not be suitable to detect signs of AD in the earliest stages before neuronal loss occurs [79]. By the time hypometabolism is measurable with FDG-PET, damage inflicted to neurons might be too severe to benefit from therapies. Additionally, increasing evidence suggests that FDG-PET shows the consumption of glucose by astrocytes, rather than by neurons and, therefore, hypometabolism can be ascribed to decreased astrocyte function [80]. Lastly, it is important to keep in mind that atypical clinical manifestations of AD may have the same pathophysiology as typical AD, but can show distinct metabolic patterns [81]. This heterogeneity in changes in metabolic patterns among the distinct AD subtypes can reduce the diagnostic accuracy of FDG-PET. Since neurodegeneration is a pathological event preceded by amyloid plaques and NFTs, biomarkers sensitive for these two events might be more suitable for the diagnosis of AD in the early stages than FDG-PET. 

### 2.3. Amyloid-PET

#### 2.3.1. Background

**Figure 4:**
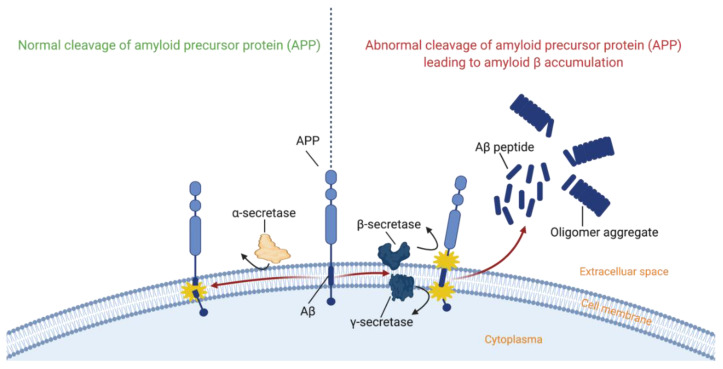
Accumulation of amyloid-β in AD. Figure adapted from Patterson et al. [89] and created with www.BioRender.com (accessed on 16 November 2020).

Many researchers believe that the first pathological event in AD is Aβ accumulation, leading to the formation of senile plaques [7,20,82,83]. However, this belief is still subject to debate [84,85]. To detect and design therapies for plaques, it is important to find out what causes plaque formation. Therefore, senile plaques were intensively studied, but due to their insolubility, the attempts to identify their composition in many studies failed [12]. Finally, in the mid-1980s, researchers were able to identify the Aβ protein as a primary component of the plaques. This protein, with an average chain length of forty-two amino residues, results from the cleavage of the larger amyloid precursor protein (APP) by β- and γ-secretase (Figure 4) [36,86]. At first, it was believed that Aβ was an abnormal protein, but the presence of Aβ in culture medium, cerebrospinal fluid (CSF) and plasma revealed that Aβ is a normal product of APP metabolism [12,83]. This understanding led to a new hypothesis: the amyloid cascade hypothesis. This hypothesis states that a dysregulation in the production and clearance of Aβ in the brain leads to the accumulation of Aβ in oligomers, protofibrils and eventually mature fibrils [87], ultimately leading to neurodegeneration and dementia. All other disease characteristics, such as the formation of NFTs out of hyperphosphorylated tau, and neurodegeneration, are seen a result of this accumulation [12,83,88]. 

#### 2.3.2. Findings

Because Aβ deposition in the brain is commonly seen as the earliest hallmark of AD, many studies have focused on identifying biomarkers that differentiate healthy controls from individuals with the first pathophysiological signals of AD. Currently, two distinct biomarkers are used to assess Aβ pathology: the concentration of Aβ42 in the CSF and amyloid-PET [1,77,90]. In this section, we discuss how PET imaging of the Aβ accumulation can contribute to the early diagnosis of AD.

##### ^11^C-PiB

In 2004, a novel ^11^C radiotracer named Pittsburgh compound-B (PiB) was applied in a study containing mild AD patients and a control group [91]. The study showed that PiB retention time was equivalent in both groups in brain regions known to be relatively unaffected by Aβ deposits. However, compared to controls, the individuals with mild AD showed considerable retention of PiB in areas known to contain substantial Aβ accumulation in AD (Figure 3). These areas included cortical areas, such as the frontal cortex and neocortex (Figure 2). The findings suggested that PET neuroimaging with PiB could provide quantitative information about Aβ deposition in living patients in early (mild AD) stages of the disease. 

Rabinovici et al. demonstrated that amyloid-PET imaging with PiB was able to distinguish AD subjects from patients with other forms of dementia, such as frontotemporal dementia (FTD) [92]. All AD subjects (7/7) had positive PiB-PET scans, while in FTB patients and healthy controls, respectively, 8/12 and 7/8 scans were negative. These findings were confirmed in a study with AD subjects and patients suffering from FTD in which the retention time of PiB in FTD patients was measured. In total, 8/10 FTD patients showed a significantly lower retention time compared to AD subjects, indicating that PiB might be a tool in differentiating FTD from AD [93]. 

In addition to differentiating between different types of dementia, amyloid-PET with PiB was able to identify the different stages of the AD continuum. In a study by Lowe et al., PiB-PET was able to significantly differentiated healthy controls from non-amnestic MCI and amnestic MIC, and AD [94]. In another study, PiB-PET clearly differentiated AD patients from MCI and healthy subjects [95]. Moreover, both studies suggested that the diagnostic value of PiB-PET increases when combined with FDG-PET, since information obtained from both techniques might be complementary. In 2014, Leuzy et al. published a paper concerning the increased PiB retention restricted to specific brain regions associated with higher levels of Aβ deposition [96]. This pattern was histological confirmed by the comparison of imaging data with immunohistochemical exams post-mortem [97,98]. 

On a molecular level, PiB is believed to bind insoluble Aβ fibrils [99]. Another study reports the strongest PiB binding to Aβ42 fibrils, followed by significant binding to Aβ42 oligomers and protofibrils [100], but compared to the fibril binding, this binding to protofibrils and oligomers is increasingly lower. Additionally, increasing evidence suggests insoluble Aβ being only a fraction of total Aβ in the brain [101] and a more prominent role of soluble protofibrils in the pathogenesis of Aβ [102]. Aβ-Pet with PiB may, therefore, be more a reflection of a fraction of insoluble Aβ than an image of total Aβ pathology in the brain. 

Over the years, amyloid-PET with PiB has emerged as the gold standard in Aβ imaging [86]. Nevertheless, PiB has its limitations and drawbacks as a radiotracer in Aβ imaging. First, the short half-life (twenty minutes) of the ^11^C isotope requires a nearby cyclotron for clinical usage [86,103]. Second, as previously mentioned, PiB has the tendency to only bind to the fibrillar form of Aβ and has low affinity for soluble oligomeric Aβ [104], while it is believed that in some genetic forms of AD, oligomeric Aβ plays a significant role in the disease manifestations [105,106]. PiB-PET might fail as a diagnostic tool in identifying these types of AD. Lastly, the selectivity of a positive Aβ scan obtained with PiB as a biomarker for AD is relatively low, because elevated PiB uptake has also been found in 30% of healthy controls without cognitive disorders [4].

##### ^18^F-Labelled Radiotracers

The short half-life of ^11^C in PiB led to the development of ^18^F-labelled radiotracers, and in 2008, the first study reported successful imaging with a fluorinated radiotracer in humans [107]. Currently, three ^18^F-labelled radiotracers for assessing Aβ deposition are approved: florbetapir, flutemetamol and florbetapen [86]. Florbetapir was the first fluorinated radiotracer and had retention ratios strongly associated with PiB [103,108]. A detailed meta-analysis into the three ^18^F-labelled radiotracers revealed no apparent differences between the diagnostic accuracy of the radiotracers [109]. However, compared to PiB, the fluorinated tracers showed higher levels of non-specific uptake in the white matter due to the more lipophilic nature of both radiotracer and white matter [110,111], resulting in more background noise [112]. Due to this extra noise, the typical white matter pattern caused by cortical amyloid plaque is lost [113]. 

#### 2.3.3. Limitations

Altogether, Aβ-PET collected the first in vivo evidence of earliest protein deposition [114]. PET has become a powerful tool in the detection of Aβ deposition and can contribute to the early diagnosis of AD. However, to fully employ its opportunities, some obstacles must be resolved. One difficulty to overcome is finding a consensus on methods to quantify amyloid-PET scans [86]. There is an urgent need for a tool to discover even the smallest Aβ deposits, and cut-off levels need to be defined in order to make studies comparable [4]. Furthermore, most studies have used radiolabeled tracers in the typical form of the AD spectrum, resulting in much knowledge about Aβ accumulation in typical AD, while the atypical, non-amnestic type has remained understudied [115]. An increasing body of evidence suggests the utility of Aβ-PET to diagnose patients with atypical manifestations of AD, such as posterior cortical atrophy (PCA) and logopenic-variant primary progressive aphasia (LvPPA) [104,116]. To maximally benefit from the diagnostic accuracy of Aβ-PET, more study into Aβ accumulation in atypical subtypes of AD is required. Lastly, the amyloid cascade hypothesis is still a hypothesis, and although Aβ deposition is an early event in the pathogenesis of AD, it may not be the direct cause of neurodegeneration and cognitive decline [86]. 

### 2.4. Tau-PET

#### 2.4.1. Background

**Figure 5:**
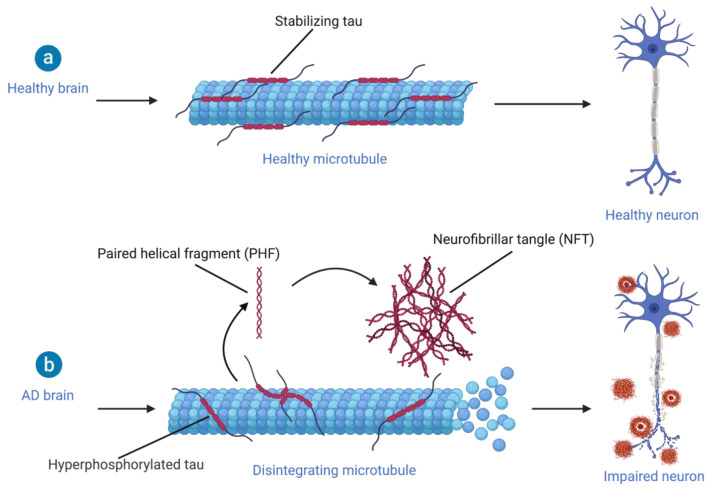
Tau protein aggregation leads to formation of neurofibrillary tangles in AD. (**a**) Role of tau protein in healthy brain; (**b**) role of tau protein in Alzheimer’s disease brain. NFT: neurofibrillary tangles; PHF: paired helical filaments. Figure adapted from “Pathology of Alzheimer’s Disease”, by BioRender.com (2020). Retrieved from https://app.biorender.com/biorender-templates (accessed on 16 November 2020).

Since multiple attempts for developing anti-amyloid drugs have failed in clinical trials, interest has shifted from treating Aβ accumulation towards development of PET radiotracers for identifying tau aggregates. This shift of interest is accompanied by the thought that tau protein aggregates are more closely related with cognitive impairment [111,117]. Furthermore, increasing evidence suggests a role for oligomeric Aβ and tau species in the early stages of AD rather than Aβ plaques and NFTs [118,119]. Tau is a microtubule-associated protein with six isoforms and is abundantly expressed in the CNS where it stabilizes the microtubules of axons (Figure 5) [120]. Several posttranslational processes can modify tau, such as acetylation, glycosylation, methylation and phosphorylation, which affect the ultrastructural conformation of tau [121]. Although normal phosphorylation of tau is required for its role in cytoskeletal plasticity during early development [122], hyperphosphorylation combined with decreased dephosphorylation leads to soluble hyperphosphorylated tau [123] that rapidly aggregates into so-called tauopathies [124]. In AD, aggregation of tau results in paired helical fragments (PHFs), and these PHFs can further accumulate into intracellular NTFs [117,123]. Even though the exact mechanism of tau aggregation is still unclear, the accumulation of tau in considered to play a major role in the neurodegenerative aspect of AD [7]. 

#### 2.4.2. Findings

**Figure 6:**
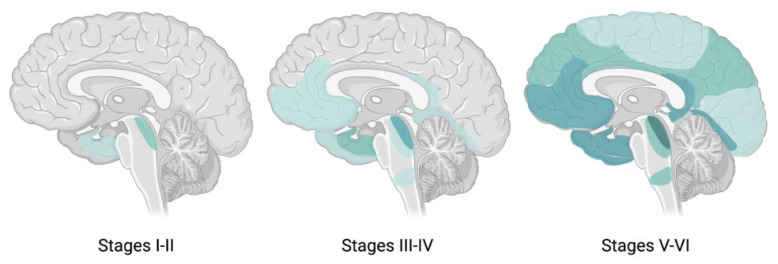
Tau spreading pattern in each Braak stage. Spreading pattern of tau throughout the brain from Braak stage I-II to stage III-IV (limbic regions) and stage V-VI (isocortical areas). Figure adapted from Goedert [145] and created with www.BioRender.com (accessed on 11 February 2021).

Just like Aβ accumulation, NFTs spread through the brain as AD progresses. This spreading pattern initiates in the entorhinal cortex (Figure 2), and as the disease progresses, NFTs spread to the limbic (stage III-IV) and isocortical (V-VI) association areas (Figure 6) [124]. However, in atypical variants of AD, the exact spreading pattern may be distinct from typical AD, and these differences in spreading patterns characterize atypical variants in early stages [125]. Although post-mortem quantification of tauopathies in the brain remains the gold standard, growing evidence suggests a role for tau-PET imaging with radiotracers in vivo for the clinical evaluation of the disease [124,126]. There are several challenges in the development of radiotracers for tau-PET. First of all, PET tracers must be able to pass the blood–brain barrier (BBB) [117]. Second, the intracellular location of NTFs poses a second barrier for the radiotracer to overcome [4,117,127]. Moreover, rapid clearance from the blood and high sensitivity are desired [117]. Since Aβ deposits and NFTs both compromise beta sheets and Aβ concentrations are remarkably higher, high affinity for tau over Aβ is required [123]. 

In AD, tau aggregates are most prominently present in the ultrastructural PHF form and therefore most attempts in developing tau-PET tracers have focused on imaging these PHFs [127]. Based on their structures, the currently available tau tracers can be divided into four groups: the nonselective tracer ^18^F-FDDNP, quinoline derivatives, pyrido−indole derivatives and PBB3 [123,124]. Computational modeling of tau fibril using cryo-EM structures of PHFs and straight filaments [128] has identified four high-affinity binding sites for tau tracers [129]. Three binding sites are buried within the core of the fibril, whereas one site is located on the surface. The next section discusses several tau PET tracers and their binding to tau at the molecular level based on this computational model. 

##### ^18^F-FDDNP

^18^F-FDDNP is a fluorinated naphthyl-ethylidene derivative and was the first tracer applied in PET imaging of tauopathy in the brain [130]. This tracer is able to bind both extracellular amyloid-β plaques and intracellular NFTs due to the presence of β sheets in these proteins [131,132]. ^18^F-FDDNP seems to favor the core sites of the tau fibril for binding [129] due to hydrophobic interactions. In addition to Aβ plaques and NFTs, ^18^F-FDDNP also binds prion plaques and is used to assess chronic traumatic encephalopathy suspicion [127,130]. Since ^18^F-FDDNP favors binding to amyloid-β over tau [127,129,132], screening of β sheet-binding small molecules was performed on a large scale to identify more suitable and specific tau tracers.

##### Quinoline Derivatives

The first selective tau PET tracers were based on quinoline and benzimidazole derivatives [111,133] and focused on the imaging of PHF tau [127]. A study by Okamura et al. synthesized three new compounds, BF-126, BF-158 and BF-170, as possible probes for in vivo tau-PET imaging in the brain [133]. The compounds showed good brain uptake combined with rapid clearance from brain tissue. Additionally, in the neuropathological exam, the three compounds were able to visualize NFTs and PHF-type neuritis, suggesting that quinoline and benzimidazole derivatives might be potential tracers for tau-PET. With these findings in mind, the search for selective tau tracers continued with the development of ^18^F-THK5105 and ^18^F-THK5117. These two compounds were developed to enhance the binding affinity to PHF-tau [7] and demonstrated binding affinity and selectivity to PHF-tau over amyloid-β in AD [134]. Similar to ^18^F-FDDNP, these two radiotracers favor the hydrophobic core sites of tau over the surface site according to the computational model [129]. Moreover, clinical PET studies revealed that these radiotracers were able to differentiate brains of AD subjects from brains of healthy controls [135,136]. A drawback of these radiotracers was the high non-negligible binding to white matter caused by the β sheet conformation of myeline. To solve this problem, ^18^F-THK5351 was developed. This new compound exhibited rapid clearance from the white matter [137]. Additionally, ^18^F-THK5351 showed higher specific binding to tau-associated regions than ^18^F-THK5117 [138]. As a result, ^18^F-THK5351 occurs to be the most promising arylquinoline radiotracer for the early detection of tau-associated pathology in AD subjects [111].

##### Pyrido−Indole Derivatives

^18^F−T808 and flortaucipir, also known as ^18^F-AV-1451 or ^18^F-T807, are both fluorinated pyrido-indole derivatives with high selectivity for tau over Aβ deposits [4,111]. Although ^18^F−T808 exhibited high tau affinity, rapid uptake and clearance, a disadvantage of this compound was de defluorination followed by bone uptake of ^18^F [139]. On the other hand, flortaucipir showed over 25-fold selectivity for tau against Aβ plaques combined with low levels of white matter uptake [4]. Moreover, the uptake of flortaucipir corresponds well with the expected spatial pattern of tau pathology in the brain of AD subjects (Figure 3) [140,141]. Furthermore, it is believed that flortaucipir binds with high affinity to all three isoforms of tau when in the classical PHF form [142], which is likely due to its high affinity for more than one binding site of the tau fibril [129]. However, flortaucipir exhibited low affinity for tau aggregates consisting of primarily straight tau filaments (Figure 5), indicating that flortaucipir might not be a suitable radiotracer in diseases other than AD. 

##### PBB3

The last group of tau-PET tracers consists of PBB3, a ^11^C-labelled radiotracer that is able to detect both AD and non-AD tauopathies [132]. The compound exhibited up to 50-fold higher binding affinity for tau than for Aβ, binds to a wide range of tau isoforms [143] and has affinity for tau at a binding site differently than other radiotracers, which might explain its wide binding range [129]. The uptake of the compounds is elevated in the hippocampus and spreads to the association cortex as disease progresses. The drawbacks of PBB3 are the usage of short half-life ^11^C in the radiotracer and the ability of its major metabolite to cross the BBB [143,144].

#### 2.4.3. Limitations

The development of novel tau tracers is an ongoing process in which several pharmaceutical companies are trying to improve the pharmacokinetics and pharmacodynamics of the tracers [7]. Compared to Aβ, the development of tau-PET tracers is still behind, and clinical validation of the tracers is required [146]. Nevertheless, tau-PET poses another neuroimaging tool for the early diagnosis of AD. 

### 2.5. Summary

Although the applications of imaging techniques in the early diagnosis of AD are on the rise, an impeccable biomarker that can diagnose AD in the earliest stage is still not available. All current techniques have their limitations (Table 1), and most importantly, there is a significant amount of protein deposition or atrophy needed for detection. Since AD is known to have decades of pathological changes before the clinical onset of disease and disease-modifying treatments may be the most beneficial before certain thresholds of protein levels or atrophy are passed, current imaging biomarkers may diagnose AD in an overly progressed stage in which therapies will inevitably fail. 

## 3. Contemporary Longitudinal Monitoring of AD with Imaging Techniques

Since AD is a progressive chronic disease with no clinical endpoint, the longitudinal monitoring of disease progression and variations in biomarker levels can give insight into the pathogenesis and prognosis of the disease [14]. In correspondence with the early diagnosis of the disease, to date, there is no consensus on the biomarkers, techniques or tests that are the most clinically relevant in monitoring the disease in the long-term. In this section, we discuss the imaging techniques and associated biomarkers that are applied in the longitudinal study of AD. 

### 3.1. Structural MRI

#### 3.1.1. Background

Whilst structural MRI might not be the most suitable technique for the early diagnosis of AD, many studies have used structural MRI to monitor disease progression, because rates of change in multiple structural measures are closely associated with changes in cognitive abilities [44]. 

#### 3.1.2. Findings

In an early study by Fox et al. in 1999, whole-brain atrophy was linked to increased disease severity [147]. Patients with untreated, probable AD were age-matched with a group of healthy controls to assess the relationship between disease severity and atrophy progression within the subject. Each individual underwent at least two MRI scans and both groups also participated in mini-mental state examinations (MMSE) on the MRI scans’ dates. The scans revealed that AD subjects had a mean rate of whole-brain atrophy of 2.4 ± 1.4% per year, while the control group had a mean loss of 0.4 ± 0.7%. Additionally, the MMSE scores demonstrated a significant difference in the mean rate of decline between AD patients and healthy individuals, indicating that rate of cerebral atrophy is strongly correlated with decline in cognitive ability. Two other studies also investigated the correlation between whole brain atrophy rates and cognitive performance and underlined the finding that whole-brain atrophy is strongly associated with cognitive decline, making cerebral atrophy an interestingly and clinically relevant biomarker for tracking AD progression [148,149]. Moreover, one of the studies implicated patients with MCI and found that a higher rate of brain atrophy per year was associated with an elevated risk of developing dementia [148]. 

In addition to whole-brain atrophy, rates of atrophy have also been evaluated in other structural regions of the brain. Cardenas et al. focused on identifying spatial patterns of brain atrophy associated with cognitive performance and possible future cognitive decline [150]. The study used deformation-based morphometry (DBM) in which every voxel is spatially normalized to a template brain. This enables the comparison between subjects with different rates of disease progression [36]. Atrophy rates in the hippocampus and entorhinal cortex (ERC) of non-demented elderly with different levels of cognitive performance were combined with several neuropsychological tests. Smaller volumes of hippocampus and ERC were strongly correlated with memory function at baseline and also predicted memory decline [150]. These results suggest that baseline volumes of these regions may predict cognitive decline due to aging, pathology or both.

**Figure 7:**
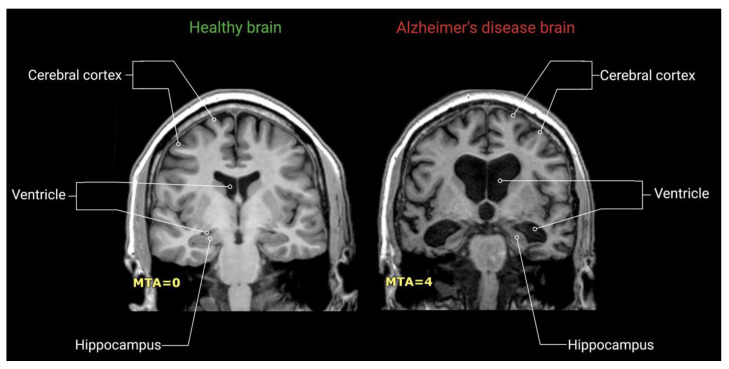
AD leads to hippocampal atrophy and ventricle enlargement. Healthy brain (**left**) versus AD brain (**right**). AD leads to decreased hippocampal volume, shrinkage of cerebral cortex and ventricle enlargement. MTA: medial temporal lobe atrophy; MTA = 0: no atrophy in medial temporal lobe; MTA = 4: severe volume loss of hippocampus. Figure created with www.BioRender.com (accessed on 14 February 2021).

Thompson et al. created maps of hippocampal and ventricular change over a longer period with a goal to visualize the spatial progression of AD and the rate of change [151]. Over time, the hippocampal volume decreased, while the ventricular volumes expanded (Figure 7). Interestingly, the spreading patterns were different between aging and dementia. Temporal horn expansion in the ventricles turned out to be a promising marker for disease progression and corresponded well with rates of cognitive decline. These results suggest that visualizations of hippocampal atrophy and ventricular expansion rates may provide a promising marker to monitor AD progression. In a study by Jack et al., the above structural MRI measures were combined and evaluated on their ability to predict disease progression [152]. A group of 160 individuals was recruited based on their profile to meet either the criteria for cognitively unimpaired, MCI or AD. All subjects had a series of MRI scans of the whole brain, hippocampus, entorhinal cortex and ventricles. Similar to the previously described studies, the change in cognitive performance was assessed with multiple tests. Over time, subjects could remain stable or shift to a more cognitive impaired group. In all brain regions, the atrophy rates were higher among subjects that converted to a more severe disease profile and supported the applicability of rates of change from longitudinal MRI measures as markers for AD progression.

#### 3.1.3. Limitations 

Although from these results, changes in atrophy rates of several brain regions may seem promising for the longitudinal monitoring of AD, these methods also have their disadvantages. Lawrence et al. found that most studies that monitor disease progression have small sample sizes with regularly below a hundred participants, probably due to high costs associated with repeated MRI measures [14]. Additionally, there is a lack of studies that implement more than one follow-up scan, and most studies have limited time between the two scans, while AD progression is protracted. Furthermore, whole-brain atrophy rates and hippocampal volume reduction are not AD-specific measures, and since MMSE is not sensitive enough to diagnose AD [153], atrophy rates and declined cognitive performance might be wrongly attributed to AD. In similarity with sMRI in the early diagnosis of AD, it is important to note that spatial patterns of atrophy differ per AD subtype [58]. In typical AD, key regions of atrophy are the hippocampus and ERC, whilst in atypical AD, such as the previously mentioned LvPPA and PCA, but also the dysexecutive/behavioral variant [154], these regions undergo slower rates of change [58,59]. It is, therefore, important to discriminate between the different types of AD before longitudinal assessments of atrophy rates are made. 

### 3.2. FDG-PET

#### 3.2.1. Background

In the previously reported study of Panegyres et al., FDG-PET was listed as a promising technique in the early diagnosis of AD and other types of early-onset dementia [60]. Although in this study, longitudinal clinical follow-up was included, this was primarily conducted as a diagnostic reference standard [74]. There are, however, other studies that have assessed the clinical relevance of FDG-PET as a tool for disease progression in AD. 

#### 3.2.2. Findings

The first study to longitudinally monitor changes in metabolism patterns with FDG-PET was a follow-up study by Drzezga et al. in 2003. MCI subjects underwent two FDG-PET scans with an interval of one year to identify typical patterns of cerebral metabolism [155]. Since patients suffering from MCI have a high risk to convert to AD within one year, FDG-PET scans of these MCI patients may give insight into the pathophysiology of AD. Converter MCI patients showed decreased glucose metabolism in the temporoparietal and posterior cingulate cortex at baseline (Figure 2). After one year, the glucose metabolism also decreased in prefrontal areas, along with a further diminished metabolism in the posterior cingulate cortex, while these regions were spared in stable MCI patients. The results indicated that metabolic change rates within one patient group can differ over time as disease progression differs. Differences in cognitive decline are correlated to different spatial patterns of decreased glucose metabolism and can be used to predict one’s risk to convert to AD. 

Fouquet et al. expanded this study by also taking into account the metabolic characteristics that distinguish converters to AD from stable MCI patients [156]. Amnestic MCI (aMCI) patients were recruited and had two FDG-PET scans with an eighteen month interval. All aMCI subjects had progressive metabolic decline over the follow-up period in the temporoparietal cortex and posterior medial parietal areas. Moreover, two medial prefrontal areas, i.e., the anterior cingulate cortex and subgenual area (Figure 2), had significantly greater decline in converters than stable aMCI subjects. This contrasts with the findings of Drzezga et al., in which lateral prefrontal regions were areas of hypometabolism. However, multiple studies support the assumption of Fouquet et al., reporting decreased metabolism in the same two medial prefrontal regions [3,157] Altogether, these findings highlight the potential of FDG-PET for the longitudinal monitoring of AD progression. 

The previously described studies did not discriminate between patients with early and late MCI. This distinction in the MCI stage has been proposed by Alzheimer’s Disease Neuroimaging Initiative (ADNI), a consortium focusing on the development of standardized biomarker procedures and use of imaging techniques in healthy, MCI and mild AD subjects [158]. The ADNI criteria classify subjects into MCI based on the scores from different tests, such as MMSE, WMS-R Logical Memory II and Clinical Dementia Rating (CDR). Classification into early MCI or late MCI is solely based on the outcome of the WMS-R Logical Memory II test, and the ADNI refers to early MCI subjects as patients that meet all the criteria for aMCI, but are in an earlier, and, therefore, less severe, point on the clinical spectrum [158,159]. With this discrepancy in mind, another research project focused on investigating differences in hypometabolism patterns and neuropsychological characteristics between early and late MCI [160]. Evaluation of the baseline scans and tests with the follow-up tests suggested that early MCI patients differ in patterns of hypometabolism and associated cognitive deficits compared to late MCI subjects. A major limitation of this study is the inclusion of only one FDG-PET scan at baseline instead of scans at every follow-up to track hypometabolism progression. There are several more studies that have identified FDG-PET as a promising tool to predict one’s risk to convert from cognitively unimpaired to MCI and from MCI to AD [161,162,163,164]. In a more recent study, patients already converted to AD were followed for three years to observe longitudinal changes in cortical glucose metabolism in amnestic and non-amnestic subjects with sporadic AD [165]. FDG-PET images at baseline demonstrated different regions with diminished glucose metabolism in amnestic and non-amnestic subjects. Similar progression patterns of metabolic reduction were observed in most regions, except for a higher rate of decline in anterior cortices in non-amnestic forms. Glucose decline progressed from anterior to posterior in amnestic patients, while in non-amnestic subjects, decline progressed along a posterior-to-anterior axis. Additionally, the non-amnestic early-onset AD patients presented more rapid and severe decline in glucose metabolism than amnestic subjects. The differences found in the spatial distribution and temporal trajectory of hypometabolism between amnestic and non-amnestic early-onset AD suggested the treatment of these two forms of sporadic AD as two separate entities. A limitation of this study was the high amount of attrition due to disease severity at baseline, which is a characteristic of early-onset AD. 

Ishibashi et al. recruited healthy individuals from an ongoing longitudinal study of cognition and aging. These controls were compared with two female subjects that were diagnosed with AD during the study and with a group of fifteen patients in the early stage of AD [166]. Female subject A had a glucose reduction rate of 9.41% over nine years, whereas female subject B’s glucose metabolism decreased with 9.07% over twelve years. In contrast, the rate of FDG reduction in the control group was 2.2% over ten years. Based on these data, the researchers estimated that diminished FDG uptake started four and two years, respectively, before clinical indications of cognitive decline in subject A and B. These differences in time between glucose hypometabolism and onset of memory loss between subject A and B are probably due to heterogeneity in the characteristics of sporadic AD and inherited AD.

Lastly, several studies have reported the usefulness of heterogeneity in hypometabolism patterns in distinguishing different variants of AD [167,168,169]. Additionally, this heterogeneity turned out to be of value in predicting progression to different forms of dementia in the prodromal MCI phase [170].

#### 3.2.3. Limitations

Although FDG-PET is thoroughly studied and seen as a robust biomarker of neurodegeneration [23], similar to other imaging techniques, FDG-PET has its limitations for the longitudinal monitoring of AD. Reduced glucose metabolism is not an AD-specific characteristic, but does also occur in a broad range of other diseases. For instance, FDG uptake is also diminished in certain brain regions after a stroke or other brain injuries [171]. Since approximately 30% of elderly people suffer from a silent infarct, lacking any clinical manifestations [172], alterations in cerebral glucose metabolism are not surprising in this aged population [173]. It is, therefore, suggested to consider FDG-PET as an independent biomarker rather than a biomarker of neurodegeneration in the “A/T/N” framework, because, as mentioned before, FDG uptake is likely to reflect the glucose consumption by astrocytes instead of neurons [80,174]. In conclusion, FDG-PET is an effective technique in monitoring glucose metabolism in the brain, but it is a tool to measure glucose uptake rather than a biomarker for longitudinal monitoring of the neurodegenerative progression in AD. 

### 3.3. Amyloid-PET

#### 3.3.1. Background

Although a fundamental role of Aβ deposition in the pathogenesis of AD is widely accepted, the relationship between plaque density and disease severity is weak [175,176]. Multiple longitudinal studies have investigated the correlation in plaque density and cognitive decline. 

#### 3.3.2. Findings

The goal of the study of Villemagne et al. was to visualize the longitudinal deposition of Aβ and to investigate the relationship between Aβ deposits and cognitive decline. Therefore, AD patients, MCI patients and age-matched healthy controls were recruited, and all subjects underwent PET imaging with PiB at baseline and follow-up. Low increased PiB retention at follow-up was found in AD and MCI patients, and in healthy controls with high retention at baseline. MCI subjects with high PiB retention had a higher chance to convert to AD than MCI patients with low PiB, and healthy controls with more PiB retention were at a higher risk to become MCI subjects than controls with low PiB. Although high levels of Aβ accumulation predicted one’s risk to convert to MCI or AD, the small increases in PiB retention only partly explained the cognitive decline, suggesting a more prominent role for other downstream factors. In a similar study by Koivonen et al., changes in Aβ burden were evaluated over a period of two years [177]. In line with the previous described study, at baseline, MCI patients had higher PiB retention compared to controls. Additionally, uptake was elevated in MCI subjects that later converted to AD than non-converters, indicating that PiB retention time can predict conversion to AD. Another study consolidated the earlier findings by stating that a positive PiB scan is a strong indication for progression of MCI into AD [178]. Interestingly, during follow-up, the PiB uptake ratio increased in non-converters, while the retention time did not increase in converters, suggesting that PiB uptake only modesty changes once converted to AD. To further assess this assumption, longer follow-up time is needed. 

**Figure 8:**
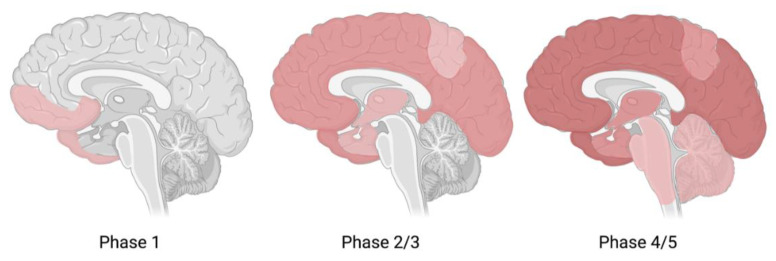
Spreading pattern of amyloid-β accumulation throughout the brain. Amyloid-β accumulation starts in frontal areas and spreads to other regions as disease progresses, leading to a plateau in amyloid-β as disease progresses. Figure adapted from Goedert [145] and created with www.BioRender.com (accessed on 11 February 2021).

Intensive longitudinal research in a group of two hundred participants revealed that Aβ deposition progresses slowly, likely to be prolonged for more than twenty years [179]. Additionally, Aβ seemed to slow down as the disease proceeded. Therefore, it is believed that as AD progresses, the Aβ accumulation will reach a plateau (Figure 1), while the cognitive decline will intensify [86]. This finding was first reported by Jack et al. in a study that modelled the temporal trajectory of Aβ deposition with PET imaging [180]. Over time, Aβ deposition followed a sigmoidal-shaped trajectory, indicating that at high Aβ load, an equilibrium is reached. In other words, Aβ accumulation precedes cognitive decline, and once a quantitative plateau is reached, the disease will become more severe. This statement is further underlined by the hypothetical model of dynamic biomarkers [77,78]. Therefore, Aβ accumulation may be a promising biomarker for predicting one’s risk to convert from cognitively unimpaired to MCI or from MCI to AD (phase 1) but may be less useful as a marker to track disease progression once a patient has established AD (phase 2-5) (Figure 8). If Aβ accumulation is no longer a dynamic marker of disease progression in the late stages of AD, it is assumed that other downstream factors are responsible for the observed associations between Aβ deposition and altered brain structures [86]. 

#### 3.3.3. Limitations

In addition to the limited value of Aβ deposition in the longitudinal monitoring of already established AD, there are other limitations associated with Aβ imaging for longitudinal monitoring of AD. For patients with already severe AD, it might be too difficult to lie still during the time needed to obtain the scans. Furthermore, PET imaging uses radiotracers, such as PiB and florbetapir. These radiotracers are known to target predominately neuritic plaques [173]. Since these types of plaques are only scarcely presented in the cerebellum, this region is often used as a reference region for many Aβ-PET studies [181]. However, multiple studies question the reliability of the cerebellum as a reference region for normalization in longitudinal Aβ deposition, since it can affect the quantitative outcome in these longitudinal studies and lead to additional variability [182]. It has, therefore, been suggested to combine multiple reference regions into one more alike to the longitudinal changes to minimize potential variations [183].

### 3.4. Tau-PET

#### 3.4.1. Background

As mentioned above, it is hypothesized that Aβ burden reaches a plateau as AD progresses [180,184], making longitudinal tracking of Aβ accumulation not the most promising biomarker for disease progression. On the other hand, tau levels slowly increase during the AD continuum with a steep increment approximately eight to nine years before disease onset (Figure 1) [21]. Therefore, longitudinal monitoring of tau accumulation may be a more powerful tool in monitoring disease progression. Moreover, increasing evidence suggests a close relationship between cognitive decline and tauopathy, making tau an interesting target for longitudinal monitoring of AD [185]. Since only recently the focus of research has shifted from Aβ deposition modifying therapies to tau associated treatment, longitudinal research with tau-PET is still in the early stages compared to decades-long studies into Aβ burden. 

#### 3.4.2. Findings

In 2015, Ishiki et al. performed a longitudinal study into tauopathy with the then novel radiotracer ^18^F-THK-5117 [186]. ^18^F-THK5117 was significantly increased in middle and temporal gyri as well in the fusiform gyrus of AD patients. Higher levels of tau load were found in patients with more severe AD compared patients with mild AD. Additionally, these tauopathies were more widely spread across the cortical regions. Furthermore, uptake of ^18^F-THK5117 was strongly associated with the rate of cognitive decline, indicating a strong relationship between neurofibrillary pathology and neurodegenerative decline. This relationship may be useful in the longitudinal assessment of disease progression and the efficacy of therapies. The assumption is underlined by a large study investigating the relationship between tau accumulation, Aβ deposition and cognitive impairment. The study included cognitively unimpaired subjects with normal Aβ levels, subjects with no cognitive impairment, but abnormal Aβ, and cognitively impaired subjects with abnormal Aβ and an amnestic phenotype [187]. The cognitively unimpaired group with normal Aβ had no detectable tau accumulation throughout the brain, whereas the unimpaired abnormal Aβ subjects had low, but significant rates (0.5% per year) of accumulation in multiple regions of the brain. This is in contrast to a study by Harrison et al. in which healthy adults with normal Aβ showed observable tau accumulation associated with brain atrophy [188]. This might be due to the different radiotracers used in the studies. The cognitively impaired abnormal Aβ subjects exhibited an increment in tau of 3% per year [187]. The accumulation rates differed only slightly from each other and during disease progression, accumulation rates increased consistently throughout the different brain areas. This indicated that tau accumulation is not restricted to one region at a time. Furthermore, the early increment in tau was not limited to the ERC, but rather widespread. Altogether, the study found that disease progression can be measured by increasing tau burden, and, therefore, tau accumulation rates may be useful as a clinical outcome for disease-modifying therapies. 

The spreading of tau throughout the brain was further studied by Cho et al. in a research study into longitudinal changes in tau accumulation in cortical regions. In contrast to Jack et al., this study reported hierarchical spreading of tau from the entorhinal cortex to other brain regions (Figure 2) [189]. This typical topography of tau accumulation is believed to initiate in the ERC and with further neuronal degeneration and more cognitive decline, it spreads to other brain areas, such as the limbic regions and association cortices [4]. Moreover, tau accumulation rates in the ERC decrease as AD progresses to higher Braak stages. 

Sintini et al. addressed the relationship between tau-PET uptake and brain atrophy in atypical AD. Interestingly, the regional patterns of tau accumulation and atrophy differed from one another in atypical AD [126]. High levels of tau accumulation were found in the frontal lobe, whereas atrophy rates were the greatest in temporoparietal areas. This difference suggested a temporal lag between tau deposition and the progression of neurodegeneration. This assumption has been previously proposed by other studies as well [114,115]. Furthermore, the research found that age has a negative effect on disease progression, since younger patients had higher rates of tau accumulation and atrophy. Lastly, there was a close relationship found between tau-PET uptake and gray matter volume. 

#### 3.4.3. Limitations

While tau accumulation is probably the most promising biomarker for disease progression, the heterogeneity of tau topography between the different AD subtypes is currently its major disadvantage [169,190]. Whereas amyloid distribution is believed to be similar, tau distribution varies between AD subtypes [59,125,191]. Additionally, each AD phenotype expresses a unique longitudinal regional pattern, and this pattern differs across the AD phenotypes [59]. It is, therefore, necessary to intensively study the dynamic patterns of AD biomarkers in atypical forms of AD in order to understand disease progression in these forms [169]. With extensive research into the different spreading patterns across the dementia spectrum, this disadvantage can become an advantage, as heterogeneity contributes to accurate longitudinal monitoring of the different AD types [190,192].

### 3.5. Summary

Over the years, great progress has been made in identifying biomarkers that reflect the disease progression of AD (Table 1). However, the distinct phenotypes of AD with corresponding heterogeneity in topographic patterns pose a challenge in the longitudinal monitoring of AD. Comprehensive research into the different subtypes of AD is needed to recognize these different patterns, and after correct identification, these pattern differences can be of additional value, such as for heterogeneity in hypometabolism patterns. Subsequently, clinical endpoints for disease-modifying therapies can be identified. 

## 4. Novel Methods, Applications of Imaging Techniques and Biomarkers in AD Research

Since there is still no effective treatment for AD and no perfect biomarker to detect AD from the earliest stages up to severe disease manifestation, much effort is put into finding novel techniques, new biomarkers and the development of new methods to apply existing techniques in the early diagnosis and longitudinal monitoring of AD. In this section, we briefly discuss new processing methods for existing techniques, possible new biomarkers that can contribute to monitoring AD and novel applications of imaging techniques in the search to successfully diagnose and monitor AD (Table 2). 

### 4.1. New Processing Methodologies for Existing Techniques

One way to increase the clinical value of already existing techniques is to improve the methods that process the obtained data. In the field of neuroimaging with MRI, much more clinical value has been obtained by using different morphometry methods for data processing. Since neuroimaging data of MRI scans are generally stored as matrices of voxels, there are several methodologies to process this type of data. Additionally, a high focus has been put on establishing new data-driven methods for the processing of PET images. 

#### 4.1.1. Voxel-Based Morphometry

The most commonly used data-driven method for T1-weighted MRI images is voxel-based morphometry (VBM), which automatically segmentizes brain tissue in white matter, gray matter and CSF [193]. It transforms T1-weighted individual brain scans into a standard reference template. Subsequently, VBM measures differences in concentrations of the different brain tissues by comparing voxels of multiple brain regions [194]. In AD, VBM is used to quantify atrophy and to automatically distinguish AD patients from MCI subjects and healthy controls [195]. 

#### 4.1.2. Deformation-Based Morphometry

Another, more biologically related method, which was previously mentioned, is DBM, in which all brain volumes are transformed into a standard template brain [36,196]. In contrast to VBM, with DBM, the high resolution of the MRI images is maintained [36]. Instead of the voxels, the deformation fields that contain information about the spatial differences of the voxels between the imaged brain and the template brain are used for statistical analysis. Therefore, DBM is more sensitive for subtle changes in brain tissue composition than VBM. Additionally, data from multiple studies, imaging equipment and research centers can be processed without bias [197]. 

#### 4.1.3. Tensor-Based Morphometry

With tensor-based morphometry (TBM), regional differences in gradients of the deformation fields that line up the images into the template brain are measured [198]. TBM can be used in a wide range of assessments, varying from the voxel level to analysis of the whole brain. Moreover, since TBM is an almost fully automated process, it is favored in large-scale MRI studies, such as clinical trials. 

#### 4.1.4. Pattern-Based Morphometry

Another type of morphometry is pattern-based morphometry (PBM), a method with its origin in VBM and DBM [199]. This data-driven method uses an algorithm based on sparse dictionary learning and is, in contrast to VBM, able to extract multidimensional patterns that characterize differences between groups, making PBM an interesting tool to compare different brain regions. Although PBM seems promising as a processing method for heterogenous disease, more research into robustness and extension to other types of neuroimaging is necessary for broad application. 

#### 4.1.5. Data-Driven Methods

As technology improves, lately, much attention has been given to using large amounts of data to build data-driven models to improve the analysis of PET images. The effect of age on certain brain regions assessed with FDG-PET, for example, has been corrected using a data-driven approach [200]. Moreover, data-driven analysis of tau PET images identified spatial patterns of radiotracer ^18^F-AV1451 signal clusters compared to pathology-based methods, suggesting an advantage for data-driven methods in evaluating radiotracer data [201]. To fully benefit from the advantages of data-driven methods in neuroimaging, extra studies comparing conventional methods with data-driven methods are necessary. 

### 4.2. Novel Implications of Imaging Techniques 

In addition to new methodologies to increase the diagnostic value of already utilized imaging techniques, novel applications of other imaging techniques are increasing. Although structural MRI with T1-weighted images is still the gold standard in AD research with MRI, other MRI sequences seem to be promising. 

#### 4.2.1. Diffusion Tensor Imaging

Diffusion tensor imaging (DTI) is an advanced type of diffusion MRI. This technique measures the displacement of water molecules in three dimensions to determine the integrity of the biological tissue [202,203]. In AD, DTI has been used to measure the integrity of brain regions by calculating the mean diffusivity [204]. Additionally, DTI demonstrated to be of value in determining the architecture of white matter [33], and multiple studies have reported the relationship between white matter integrity and disease severity, suggesting the inclusion of white matter degeneration as a pathological biomarker of AD for early diagnosis [205,206,207]. In order to exploit the full potential of DTI as a diagnostic tool in AD, the exact relationship between disease severity and white fiber tracts has to be explored [203].

#### 4.2.2. Functional MRI

Functional MRI (fMRI) is an imaging technique that gives insight into the functional integrity of brain networks that support several cognitive domains in a non-invasive manner [23,208]. fMRI uses the blood-oxygen-level-dependent (BOLD) signal to measure the synaptic activity of neurons. fMRI can be used in two manners: resting state (rs) fMRI, which measures changes in BOLD signals during inactivity, or task-related fMRI in which patients perform several cognitive tasks [33]. Since severely impaired patients may be too limited to perform these tasks, rsfMRI may be more feasible to monitor disease progression in later stages [23]. Although fMRI has been demonstrated to be of clinical value in studying the default mode network [209,210,211,212], clinical use of fMRI is not widely supported due to limitations, such as low signal and noise [202]. 

#### 4.2.3. Optical Coherence Tomography

Another interesting imaging technique to be applied in AD research is optical coherence tomography (OCT), but to date, there is no consensus on the employment of this technique. In recent years, pathological changes in the retina have been linked to AD [213]. These changes include Aβ plaques, thinning of the retinal nerve fiber layer (RNFL), ganglion cell loss and decreased vessel density. Since OCT is a non-invasive, fast and inexpensive technique [214], multiple studies have investigated the beneficial value of OCT in AD research. Although accumulation of Aβ in the lens, analysis of RNFL thickness and ganglion cell loss are proposed as diagnostic tools for AD [215,216,217,218], the reliability of these markers is still questioned due to possible other underlying diseases that cause these pathological changes, such as glaucoma. Nevertheless, the feasibility and cost effectiveness of OCT make it an interesting imaging technique to further investigate for applications in AD.

### 4.3. New Biomarkers

For many years, the focus of AD research has been on atrophy, glucose metabolism and imaging of Aβ deposition and tau burden. However, since none of these biomarkers stands out as a faultless biomarker for the diagnosis of AD and disease progression, research focuses on identifying novel biomarkers that reflect the progression of AD. Over the years, several new biomarkers have been introduced.

#### 4.3.1. Synaptic Vesicle Glycoprotein 2A

One marker suggested to be of clinical value in AD is synaptic vesicle glycoprotein 2A (SV2A), which reflects the synaptic density [111]. This protein is located in the cell membrane of secretory vesicles, and since SV2A is ubiquitously expressed throughout the brain, lower levels of SV2A may be a promising biomarker of synaptic loss in AD. ^18^F-UCB-J is a PET radiotracer considered to be sensitive for synaptic loss, because altered uptake of ^18^F-UCB-J in the gray matter was correlated to altered expression of SV2A and lower synaptic density [219,220]. Although these results seem promising, large scale validation of this and other radiotracers is necessary to further exploit the clinical possibilities of SV2A in AD [9].

#### 4.3.2. Receptor for Advanced Glycation End Products

There is increasing evidence that the receptor for advanced glycation end products (RAGE) regulates the neurotoxicity of Aβ in AD [221]. The binding of RAGE to Aβ results in the release of reactive oxygen species that contribute to the formation of senile plaques and NFTs. Moreover, RAGE levels are significantly higher in AD subjects than in cognitively healthy controls [222]. Therefore, it has been suggested that in the early stages of AD, RAGE is a potent biomarker. ^11^C-FPS-ZM1 is a radiotracer for PET imaging of RAGE in the brain [223]. Since RAGE overexpression is believed to precede the formation of Aβ plaques, PET imaging of RAGE with ^11^C-FPS-ZM1 may be a powerful tool in the early diagnosis of AD [221].

#### 4.3.3. Iron

Excessive accumulation of iron in specific brain parts is increasingly related to AD [224]. Although iron is required for maintaining homeostasis and plays a key role in many biological processes, abnormal accumulation of iron in subcortical and deep gray matter nuclei has been associated with AD [225]. Multiple studies have used quantitative susceptibility mapping (QSM) to quantify the local magnetic susceptibility derived from MRI images caused by deposits containing both Aβ and iron [224,226,227]. QSM application in the detection of iron has been demonstrated to be of clinical value in assessing the relationship between Aβ accumulation and iron burden [226]. Therefore, the detection of iron with QSM may be of potential aid in imaging Aβ in the early diagnosis of AD [227].

## 5. Conclusions and Future Perspectives

In this review, we focused on the applications of imaging techniques in the early diagnosis and longitudinal monitoring of AD. AD is a neurodegenerative disease in which pathological changes occur decades before disease manifestation. The disease is characterized by the formation of senile plaques, NFTs and subsequent synaptic loss and neurodegeneration. Although AD affects a major part of the population worldwide, to date, there is no therapy to cure AD. Since disease-modifying therapies may be the most beneficial in early stages of the disease, it is important to diagnose AD as early as possible. Additionally, longitudinal monitoring of disease progression is crucial to gain a better understanding of the pathogenesis and to set clinical endpoints for potential treatment. To date, several biomarkers have been proposed for the early diagnosis and longitudinal monitoring of AD, but all these biomarkers have their limitations regarding specificity, reliability and sensitivity.

Aβ deposition is among the earliest hallmarks of AD, but to date, there is no consensus on exactly how Aβ accumulation contributes to the pathogenesis of AD. Moreover, detailed study into Aβ over time has revealed that Aβ levels reach an equilibrium, making Aβ a questionable biomarker for monitoring disease progression. Tau accumulation, on the other hand, is believed to be more biologically related to the symptoms associated with neurodegeneration in AD. Imaging studies with tau PET-tracers have demonstrated promising results, but compared to Aβ-PET imaging, large-scale validation of these tracers must be performed to make tau-PET imaging a reliable tool in AD. Moreover, longitudinal studies into different phenotypes of AD revealed heterogeneity in the topographic patterns of tau accumulation throughout the brain. This heterogeneity can be of additional value, but first, more detailed study in these different tau spreading patterns is required. More general imaging techniques, such as FDG-PET and structural MRI, have been applied in AD research, but these techniques measure rather more common pathological changes than AD-specific characteristics. Brain atrophy, measured by structural MRI, is not restricted to AD pathology and is only detectable after a substantial amount of neurodegeneration. FDG-PET is used to measure the glucose uptake in the brain. Since synaptic loss in AD leads to hypometabolism, decreased glucose uptake is associated with AD. However, decreased glucose metabolism is not restricted to AD but can also occur after strokes and brain injury. Furthermore, an increasing body of evidence suggests that glucose uptake reflects astrocyte function rather than neuronal function.

Altogether, to date, there is no perfect biomarker to detect AD in the early stages and to monitor disease progression over time. Furthermore, these biomarkers rely on neuroimaging techniques that require high-quality and expensive machinery, making them infeasible for large-scale examinations of greater populations.

Hence, comprehensive and in-depth research into AD is crucial in the early diagnosis and longitudinal monitoring of AD. Since tau-PET appears to be the most promising tool for the diagnosis and tracking of disease progression, the field of research should focus on the validation and development of existing and new tau radiotracers. Furthermore, detailed research into new applications of other imaging techniques is necessary to overcome the limitations in the extensive scanning of large populations. Lastly, current tools require relatively high levels of protein accumulation or neurodegeneration to be detectable. Since higher levels are associated with higher disease severity and lower beneficial potential of therapies, identifying novel biomarkers that reflect the pathogenesis of AD in the earliest stages is essential for the development of disease-modifying therapies. 

## Figures and Tables

**Table 1:** Advantages and limitations of imaging techniques currently used in early diagnosis and longitudinal monitoring of AD.

Technique		Early Diagnosis	Longitudinal Monitoring
Structural MRI	Advantages	Powerful in predicting volumes	Changes in atrophy closely related to changes in cognitive abilities High atrophy rates predict cognitive decline
		MRI scanners widely available Safe	
	Limitations	Direct observation of Aβ plaques or NFTs not possible	Findings based on small population sizes and limited number of scans
		Decreased hippocampal volume not AD-specific measure Atrophy patterns differ among AD subtypes	
FDG-PET	Advantages	Extensive research led to an FDG-PET endophenotype usable for comparison Highly sensitive and specific	Differences in metabolism patterns able to predict risk to convert to AD Diminished FDG uptake precedes clinical manifestation Heterogeneity in topographic progression of reduced metabolism may predict AD variant
	Limitations		Reduced glucose metabolism caused by other diseases or injuries
		Rather reflection of glucose consumption by astrocytes Invasive due to injection and radiolabelled tracer Expensive and not widely available	
Amyloid-PET	Advantages	Aβ plaques seen as earliest hallmark of AD Retention time of radiotracers matches spreading pattern of Aβ plaques	PiB retention time able to predict conversion from MCI to AD
	Limitations	Exact role of Aβ accumulation in AD still unknow Elevated PiB uptake also found in healthy controls No standard method for quantifying Aβ plaques Not much known about Aβ accumulation in atypical forms	Weak correlation between Aβ deposition and disease severity Aβ accumulation stabilizes in later stages of AD Choice of reference region subject to debate
		Invasive due to injection and radiolabelled tracer	
Tau-PET	Advantages	Tau accumulation believed to be closely related to cognitive impairment Radiotracer uptake matches spreading pattern of tau Radiotracers have high affinity for PHF tau	Strong relationship between neurofibrillary pathology and neurodegeneration Accumulation rates consistently increase throughout the brain
	Limitations	Most tracers low affinity for straight filaments	High level of heterogeneity in tau topography between AD subtypes
		Still new field of research Invasive due to injection and radiolabelled tracer	

**Table 2:** Summary of novel strategies in AD research.

**Methodology**	Voxel-based morphometry	Automated segmentation of brain tissues Comparison of voxels to measure concentration differences
	Deformation-based morphometry	Transformation of all brain volumes to standard template brain Statistical analysis of deformation fields
	Tensor-based morphometry	Uses regional differences in gradients of deformation Favored in large-scale MRI studies
	Pattern-based morphometry	Able to extract multidimensional characteristics More research necessary for broad application
	Data-driven methods	Large amounts of data can improve image quality More comparison between conventional and data-driven methods necessary
**Imaging technique**	Diffusion tensor imaging	Measures displacement of water in three dimensions Needs more research to exploit full potential
	Functional MRI	Uses BOLD signal for synaptic activity of neurons Not widely supported due to several limitations
	Optical coherence tomography	Non-invasive and cheap technique to assess effect of AD in the eye Reliability still question of debate
**Biomarker**	SV2A	Reflects synaptic density in brain Large scale validation necessary for broad application
	RAGE	Believed to regulate toxicity of Aβ Potentially powerful biomarker in early diagnosis
	Iron	Relationship between Aβ and iron accumulation Detection of iron with QSM promising tool

SV2A: synaptic glycoprotein 2A; RAGE: receptor for advanced glycation end products; QSM: quantitative susceptibility mapping.

## Abbreviations

AD	Alzheimer’s disease
NFT	Neurofibrillary tangle
Aβ	Amyloid-β
MRI	Magnetic resonance imaging
PET	Positron emission tomography
MCI	Mild cognitive impairment
CNS	Central nervous system
FDG-PET	Fluorodeoxyglucose positron emission tomography
APP	Amyloid precursor protein
CSF	Cerebrospinal fluid
PiB	Pittsburgh compound-B
FTD	Frontotemporal dementia
PHF	Paired helical fragment
BBB	Blood-brain barrier
MMSE	Mini-mental state examination
DBM	Deformation-based morphometry
ERC	Entorhinal cortex
MTA	Medial temporal lobe atrophy
aMCI	Amnestic mild cognitive impairment
ADNI	Alzheimer’s Disease Neuroimaging Initiative
VBM	Voxel-based morphometry
TBM	Tensor-based morphometry
fMRI	Functional magnetic resonance imaging
BOLD	Blood-oxygen-level-dependent
rsfMRI	Resting state functional magnetic resonance imaging
DTI	Diffusion tensor imaging
OCT	Optical coherence tomography
RNFL	Retinal nerve fiber layer
SV2A	Synaptic vesicle glycoprotein 2A
RAGE	Receptor for advanced glycation end products
QSM	Quantitative susceptibility mapping
